# Automated *C*. *elegans* embryo alignments reveal brain neuropil position invariance despite lax cell body placement

**DOI:** 10.1371/journal.pone.0194861

**Published:** 2018-03-28

**Authors:** Peter Insley, Shai Shaham

**Affiliations:** Laboratory of Developmental Genetics, The Rockefeller University, New York, NY, United States of America; Brown University, UNITED STATES

## Abstract

The *Caenorhabditis elegans* cell lineage is nearly invariant. Whether this stereotyped cell-division pattern promotes reproducibility in cell shapes/positions is not generally known, as manual spatiotemporal cell-shape/position alignments are labor-intensive, and fully-automated methods are not described. Here, we report automated algorithms for spatiotemporal alignments of *C*. *elegans* embryos from pre-morphogenesis to motor-activity initiation. We use sparsely-labeled green-fluorescent nuclei and a pan-nuclear red-fluorescent reporter to register consecutive imaging time points and compare embryos. Using our method, we monitor early assembly of the nerve-ring (NR) brain neuropil. While NR pioneer neurons exhibit reproducible growth kinetics and axon positions, cell-body placements are variable. Thus, pioneer-neuron axon locations, but not cell-body positions, are under selection. We also show that anterior NR displacement in *cam-1*/ROR Wnt-receptor mutants is not an early NR assembly defect. Our results demonstrate the utility of automated spatiotemporal alignments of *C*. *elegans* embryos, and uncover key principles guiding nervous-system development in this animal.

## Introduction

The somatic cell lineage of the nematode *C*. *elegans* is essentially invariant between individuals. Thus, for example, *C*. *elegans* hermaphrodites contain 302 neurons, each identifiable by name, with a lineal history traceable back to the zygote. Constancy in lineage could dictate reproducibility in cell placement and morphology, as has been previously suggested [1,2]. Alternatively, cell position and shape could be governed by other imperatives. How lineage relates to cell positioning/morphology is of particular interest in the nervous system, where neurons must interact with specific partners to form circuits generating behavior.

Assessing reproducibility of cell shape and positioning during *C*. *elegans* development requires precise alignments of developing embryos. However, embryos are usually imaged in arbitrary orientations and exhibit variable developmental movements [2–4]. Thus, quantitative cell position/shape comparisons must correct for such bulk differences in animal configuration. Furthermore, since embryogenesis lasts 14 hours at 2°C, and important developmental events occur on minute time scales, temporal alignments must be ensured.

Manual alignments of high-resolution movies capturing *C*. *elegans* embryogenesis are tedious and low throughput, and existing computational methods can extract embryo features, but have not been used for automated alignments [3,4]. To this end, we developed a fully automated method for spatiotemporal alignment of *C*. *elegans* embryos from pre-morphogenesis (~50–100 cells) to the onset of muscle twitching [1]. Fiduciary markers were previously used for alignments in a number of settings [5,6,7,8]. We used this approach, together with organism-wide markers [2,9] in our studies. We employ a pan-nuclear marker for staging, sizing, and initial embryo orientation, and a sparsely-distributed nuclear marker, defining fiducial points, to track developmentally variable rotations and translations. The method uses increasingly accurate registration steps that avoid misleading local alignment solutions, to align test embryos onto a reference. Some of the strategies we use are reminiscent of those employed in [10], studying mouse embryonic development, although the details are quite different. An overview of our method is found in S1 Fig.

To examine the efficacy and utility of our algorithm, we studied the development of the *C*. *elegans* brain neuropil, the nerve ring (NR). Approximately 180 *C*. *elegans* neurons send processes into the NR, where most synapses are found [11,12]. While neurons are added to the NR throughout development [11], the structure begins assembly between the bean and comma stages of embryonic development, slightly after embryonic morphogenesis onset [13]. The NR is initially demarcated by processes of CEPsh glial cells with radial-glia-like features, which form a structure that later circumscribes the pharynx. These glia guide axons of 5 bilateral pairs of sublateral (SubL) pioneer neurons to form a NR primordium consisting of ~10 processes. Glia and pioneer neurons then together direct NR entry of follower axons [13].

We used a custom-built single-plane-illumination microscope [14,15] to collect 34 movie stacks of early NR assembly in developing embryos expressing green-fluorescent protein (GFP) in SubL neurons. Remarkably, embryo alignments using our algorithm reveal that while NR SubL axons are positioned to within ~1 μm in different embryos, SubL cell body positions are much more loosely aligned. Unlike SubL neurons, one neuron we imaged, ALA, whose cell body resides near the NR anterior apex, shows little positional variation. Thus, NR and ALA cell-body positions are under precise control, whereas SubL neuron cell-body positions are not under tight selection. The substantial variability we observe for SubL cell body placement is surprising, given that *C*. *elegans* development has been suggested to be invariant [1,2].

Having characterized the fidelity of early nerve ring formation, we used our method to align embryos bearing mutations in *cam-1*/ROR, a receptor-tyrosine-kinase gene required for NR positioning in larvae [16,17]. These studies uncover previously-unappreciated defects in *cam-1* mutants, including formation of a giant NR, associated with loss of animal viability. Furthermore, we show that in surviving animals, initial NR positioning is not perturbed, suggesting that *cam-1* likely functions for later maintenance of NR placement.

Our studies demonstrate the utility of automated embryo alignments for quantitative studies of cell shapes and positions. Since our alignment procedure is generic, it can be extended to other reporter combinations in *C*. *elegans*, as well as to other model systems in which a pan-nuclear marker and an asymmetric fiduciary marker can be identified.

## Results

### Challenges in aligning *C*. *elegans* embryos

A straightforward way to align two objects is to identify fiducial markers common to both, and then rotate and translate one with respect to the other, until the markers overlap. Thus, in principle, to align two *C*. *elegans* embryos, each would express a fluorescent reporter labeling a common set of cells, and rigid transformations would then be used to overlap these labels. In practice however, this simple idea is remarkably challenging. Since embryos are not rigid objects and embryonic development involves dynamic cell movements, it is impossible to identify fiducial cells whose positions remain constant in time. It is also impossible to keep the number of fiducial cells constant over time, as labeled cells in early embryogenesis divide. Furthermore, most gene reporters display dynamic expression patterns; thus, embryos must be temporally aligned so that identical time points are compared. Even then, positions and label intensity of fiducial labels may vary between embryos as a result of reporter expression differences. Embryo orientation will also affect signal, as spherical aberration and scattering processes will reduce signal from deeper layers. Thus, perfect alignments are generally unattainable, and a best fit should be sought. Often, however, multiple fits with similar alignment scores can be found, and the relevant solution must be identified. Finally, embryos exhibit bulk rotations that vary in magnitude, and are imaged at different initial orientations. Thus, the same alignment parameters cannot be used for each embryo and each time point.

Below, we describe our procedures for addressing these challenges. Embryo image stacks for alignments were acquired on a custom-built single-plane-illumination microscope (Methods) at 20°C for 20 consecutive 10-min intervals (T1-T20) starting within 20 minutes of the first cell division and through the embryonic ball stage [1], to determine embryo shape and temporal staging. Imaging frequency was then increased during morphogenesis, and 125 consecutive images (T21-T145) collected at 2-min intervals, until muscle twitching onset. One embryo was arbitrarily selected as a reference to which test embryos were aligned. An overview of the alignment procedure is presented in S1 Fig.

### The imaging strain and image segmentation

**Fig 1:**
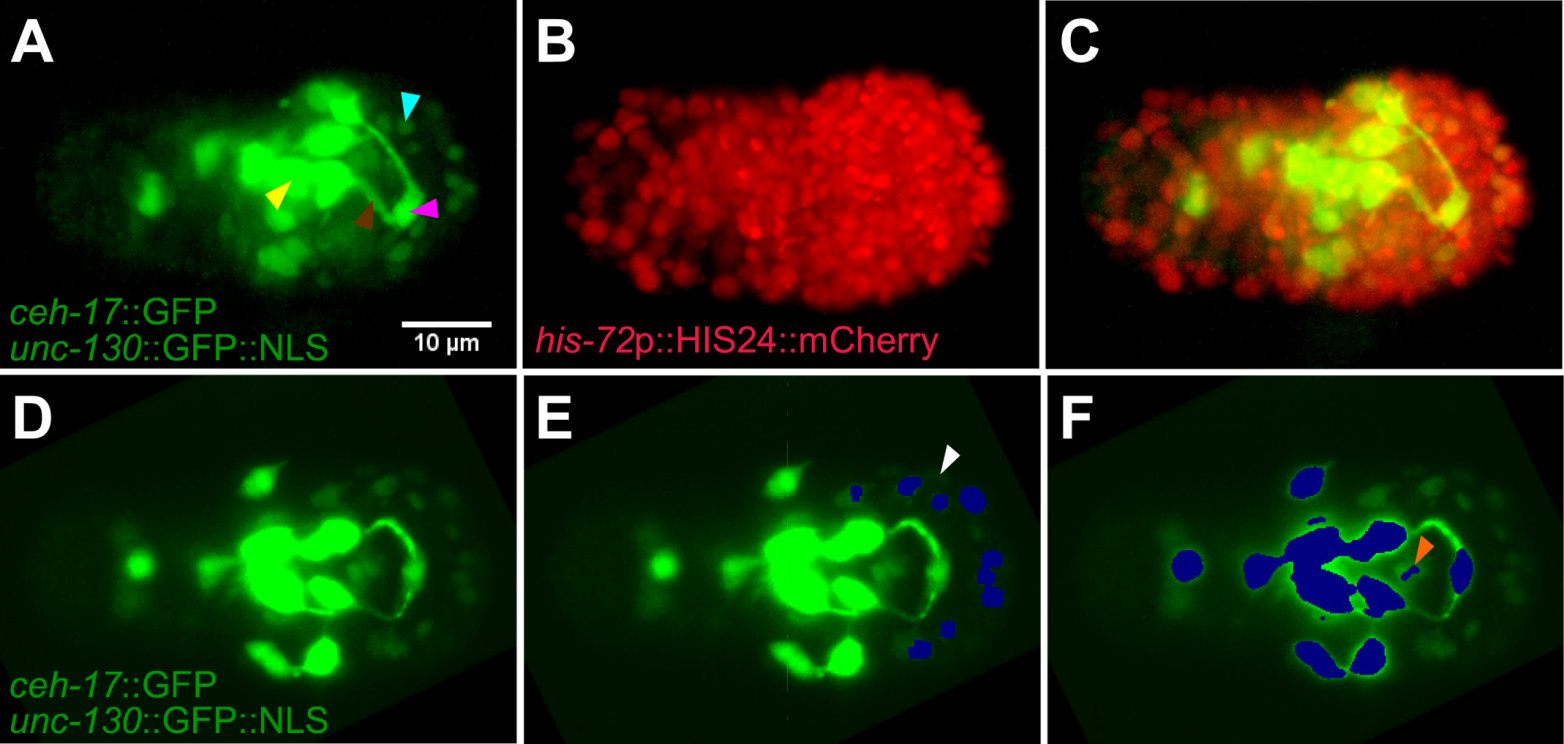
Imaging strain reporters and GFP segmentation. **A-C)** Embryo, just before twitching, expressing indicated fluorescent reporters for alignments and for visualization of the nerve ring and sublateral neuron cell bodies. **A**) Cyan arrowhead, *unc-130*::GFP-labeled nucleus. Yellow arrowhead, sublateral neuron cell body. Brown arrowhead, nerve ring. Magenta arrowhead, ALA neuron. **B)** mCherry channel of same embryo. **C**) Merge of **A** and **B**. **D-F**) Segmentation of GFP signals on a test embryo not used for training the classifier. **D**) Raw image. **E**) Blue, segmented *unc-130*::GFP nuclei. Segmentation is restricted to anterior half of embryo outside a central cylinder surrounding the nerve ring. White arrowhead, false negative. **F**) Blue, *ceh-17*::GFP sublateral neuron cell body segmentation. Orange arrowhead, false positive.

We used two reporter transgenes for alignments: *his-72*p::HIS-24::mCherry [18], expressing mCherry in all nuclei from the 50-cell stage onward; and *unc-130*p::NLS::GFP [19], expressing GFP in nuclei of anterior cells starting at the 100 cell stage (4–40 nuclei, depending on stage), and in 4 posterior nuclei. To visualize NR development, a *ceh-17*::GFP cytoplasmic reporter, labeling the SIAVR/L, SIADR/L, and SIBVR/L SubL neurons, and the ALA, RMED, DA8, and DB5 neurons was used [20–22] (Methods; Fig 1A–1C; S1 Movie). This reporter was not used for alignments.

To facilitate alignments of embryos between consecutive time points, and of test embryos onto the reference (see sections below), we sought to automatically identify and distinguish fluorescent signals produced by each reporter protein.

To assign mCherry nuclei, we used the StarryNite program, previously designed for segmenting *C*. *elegans* nuclei from image stacks [23]. By tuning the parameters that govern the probability of segmenting a nucleus from a given region, such as “intensity threshold”, to generate a low number of false positives (at the expense of producing some false negatives), we automatically generated a list of nuclear centers for the first 40 time points of each movie, without the usual manual curation (Methods). Parameter thresholds were adjusted until nuclear segmentation outside of the eggshell was not observed in an early test population (not used for further analysis).

GFP segmentation was more complicated, since *unc-130*p::NLS::GFP nuclei, as well as *ceh-17*::GFP neuronal cell bodies and processes, are labeled with this protein. Attempts to differentiate between these features using brightness failed to separate *ceh-17* neurites and *unc-130* nuclei. However, using the machine-learning segmentation program Ilastik [24], we generated a classifier that distinguished between these signals (Fig 1D–1F; S2 Fig; Methods). The classifier was obtained by training the program on five embryos, including the reference embryo, on four time points per embryo during the fast imaging period (T21-T145). The classifier was trained to reduce cross-assignments of *unc-130*p::NLS::GFP and *ceh-17*::GFP at the expense of a high rate of false-negative assignments (Panels B-C in S2 Fig), apparently keeping thresholds for nuclear detection within narrow bounds. This procedure was facilitated by only considering *unc-130*p::NLS::GFP signal outside a cylindrical region surrounding the NR in the anterior half of the embryo, and by restricting segmentation to the largest contiguous objects, after use of a Gaussian blurring filter that fills in gaps in objects, thereby reducing signal from disconnected single-pixel-scale objects (Methods).

### Temporal alignment of developing embryos

**Fig 2:**
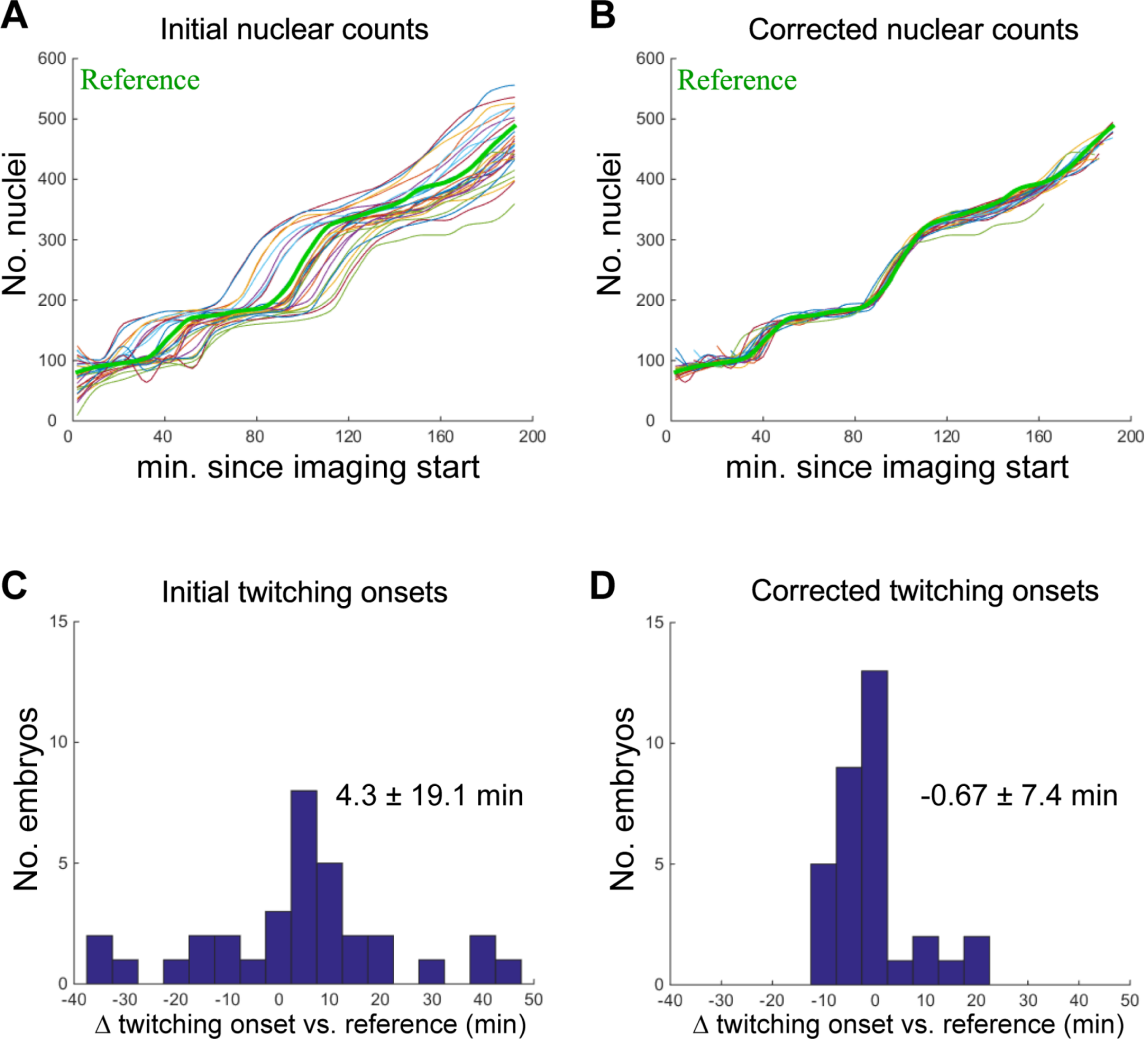
Temporal alignments of embryos. **A,B**) Bold green curve: reference embryo. Other curves, individual test embryos. **A**) Raw nuclear counts over time. **B**) Nuclear counts over time after implementation of temporal correction algorithm. **C,D**) Onset of twitching, relative to reference embryo without (**C**) and with temporal alignment (**D**). Mean and SD of data are indicated.

Variability in developmental rates and imaging start times must be addressed to ensure alignment of a test embryo movie to the reference movie. To that end, we collected Starry-Nite segmentation estimates of the number of nuclei per time point over the 20 consecutive frames of slow imaging (T1-T20). The number of nuclei between frames was then interpolated using piecewise cubic hermite polynomials [25], adding four additional nuclei number estimates between consecutive frames, for improved resolution in matching. The resulting temporal distribution of nuclei number was shifted to match that of the reference embryo by a linear time offset (Fig 2). The offset was chosen to minimize the summed square difference of nuclei numbers across corresponding time points. The root mean square error over all embryos and time points, measured with respect to the reference embryo, and normalized for the number of overlapping time points before and after offset, is 39.2 nuclei prior to offset application, and 10.0 nuclei after. The mean offset across all test embryos was -5.0 min. As seen in Fig 2B, time courses of different embryos display similar characteristics, and appear aligned to within a few minutes, suggesting that further temporal adjustment, such as multiplicative scaling (e.g. [26]), may be unnecessary.

To confirm this, we timed the onset of the first instance of muscle twitching. While the standard deviation in twitching times prior to offset application was 19.1 min, it was reduced to 7.4 min following offset implementation (Fig 2C and 2D). This observation supports the idea that our temporal alignment is accurate to within a few minutes in the time up to twitching, sufficient to track most known developmental events. Surprisingly, our results also suggest that onset of twitching is a precisely reproducible developmental time point, an observation that to our knowledge had not been quantitatively demonstrated before. Correlation of calculated temporal offsets with twitching time was found to be similarly precise to correlation of twitching times with individual cell division plateaus during development (r = .94 for temporal offsets; r = .90 for each of the two plateaus which could reliably be extracted from the data; Methods).

### Defining an embryonic long axis

**Fig 3:**
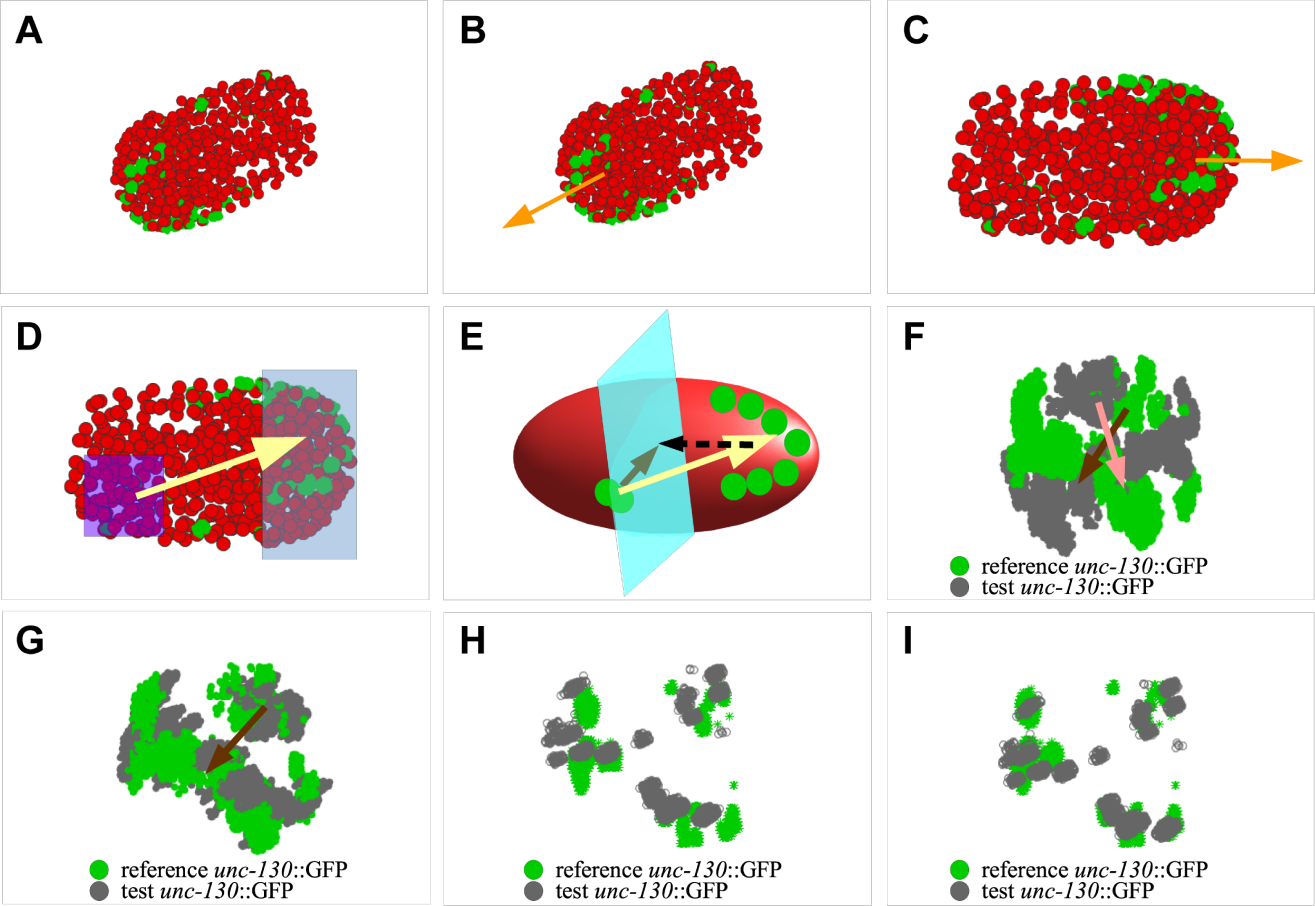
Defining a universal coordinate system for alignment of test and reference embryos. Green, *unc-130*::GFP segmentation. Red, mCherry segmentation. **A**) Raw segmented test embryo. **B**) Axes of the embryo determined using convex hull of mCherry segmentation. Long axis, orange. **C**) Using information in **B**, embryo is centered, aligned to the camera axis, and rescaled. **D-F**) An axis perpendicular to the long axis is defined by generating a vector (yellow arrow) pointing between clusters of *unc-130*::GFP expression (**D**), and projecting onto a plane perpendicular to the long axis (**E**). Solid olive-green arrow, perpendicularly projected vector. Dashed arrow, direction of projection. **F**) Raw overlays of *unc-130*::GFP segmentation of a test embryo (gray) and the reference (green), prior to establishing axes perpendicular to the long axis. This step uses a relaxed segmentation threshold. **G**) Overlay of same segmentation as in **F**, after rotation to align the projection vectors found in **E**. **H-I**) Fine correction for rotation transform in **G**. **H**) Segmentation as in **G**, with a tighter segmentation threshold, at a time point twenty minutes earlier. **I**) Point clouds from **H** are matched using CPD-register, to provide fine corrections.

To spatially align embryos that have been temporally aligned, we sought to define coordinate axes for each embryo that could then be brought computationally into correspondence with a similar coordinate system for the reference embryo. To define an axis corresponding to the anterior-posterior (long) axis in early embryos, we used the positions of the mCherry nuclei on the surface of each pre-morphogenesis embryo as an indicator of overall embryonic shape (Fig 3). The surface nuclei were defined by taking the convex hull of all nuclei (i.e., the smallest convex surface containing all nuclei). We then defined a uniform-density volume by filling the interior of the convex hull, producing a model of the embryo volume, and calculated the principle moments of inertia and centroids of the resulting distribution (Fig 3A–3C). The axis corresponding to the smallest principle moment (P1 axis) was placed along the camera *x*-axis (Fig 3C, orange arrow). We averaged our results over 16 time points (T15 –T30) for the reference embryo. This time period was chosen as it corresponds to the period (ball-bean stage) during which an embryo grows to its maximum extent in the eggshell without elongation drastically changing its geometry [1,3]. The same developmental time period was used to average P1 axis positions in test embryos.

Subsequently, the anterior end of the embryo was identified from the *unc-130*p::NLS::GFP segmentation, using the observation that overall *unc-130*p::NLS::GFP expression is stronger anteriorly (Fig 3B and 3C). Thus, embryos are positioned with their long (P1) axis on the camera *x*-axis, with anterior to the right.

### Adjustments for embryo size variability

While the spatial orientation of the coordinate axes is not affected by embryo size, the scale of these axes is different in embryos of different sizes, and this must be corrected before alignment proceeds. To do so, the moments of inertia vectors of each ball-stage test embryo were compared to those of the reference embryo, and multiplicative scaling factors that brought these into correspondence were determined (Fig 3C; Methods). These scaling factors were then used to transform each set of test embryo images to match the reference embryo scale. The short-axis moments are similar at this stage, as the embryo is rotationally symmetric, thus only two scale factors, for length and width, were necessary (Methods). We found that required embryo size adjustments are small, with average length and width adjustments of 2.9% (max = 9.3%), and 1.3% (max = 4.8%), respectively. Scaling parameters calculated using a minimum-volume ellipsoid method [2,27] correlated highly with those obtained by the moment-of-inertia scaling procedure (r = .76 for width, r = .91 for length, p<10^−6^ by Z test), suggesting that the adjustments likely reflect real differences in embryo sizes. Nonetheless, the absolute changes are small (around 0.5 μm width and 1.5 μm length on average for a 25 x 50 μm embryo).

### Defining time-invariant perpendicular coordinate axes

Moments of inertia vectors could not be used to define unambiguous coordinate axes around the P1 long axis, as the early embryo exhibits high rotational symmetry around this axis. However, we noticed that the *unc-130*p::NLS::GFP marker is asymmetrically expressed in a group of posterior nuclei on the ventral side in a period around T20-T30 in the reference embryo and in corresponding periods in the test embryos. For each embryo, we therefore constructed a vector, originating at the average posterior *unc-130*p::NLS::GFP position and terminating at the average anterior *unc-130*p::NLS::GFP position, and computationally projected this vector onto a plane perpendicular to the long axis (Fig 3D and 3E). This operation defines two coordinate axes, P2 and P3, perpendicular to the long axis, which are approximately consistent between embryos. While individual cell movements occur prior to morphogenesis, bulk embryonic rotations within the eggshell do not appear to take place during this time period. Thus, for each embryo, the coordinate system we established is time invariant at these early stages.

**Fig 4:**
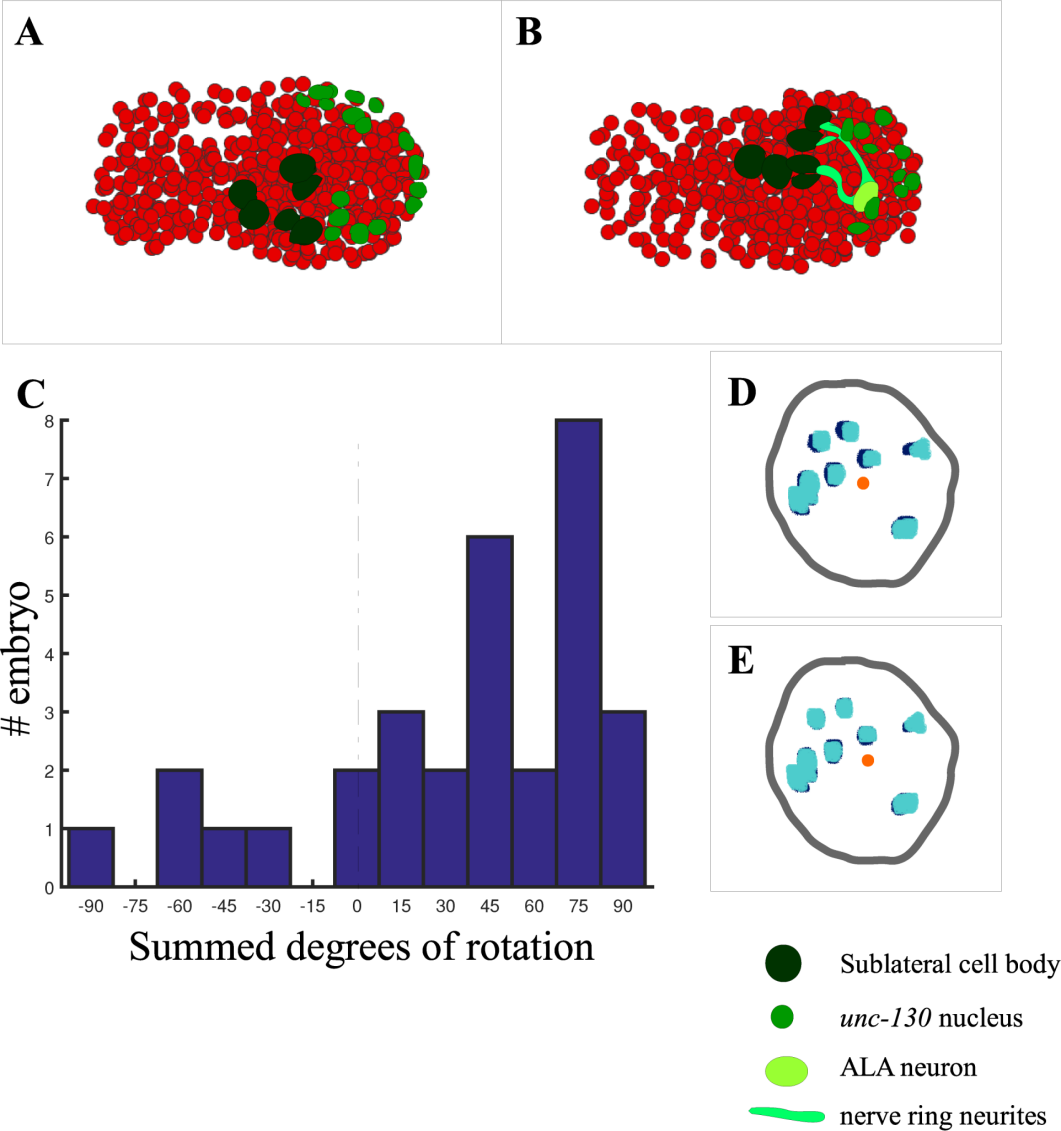
Correcting internal embryo rotations. Internal coordinate systems are established for individual embryos by correcting rotations about the AP-axis throughout development, by point-matching segmented nuclei against one another between time points. **A**,**B**) Segmentation of an embryo before (**A**) and after (**B**) an internal rotation (and concurrent neurite outgrowth). **C**) Summed degrees of internal rotations, calculated using the rotation correction method described in the text, in degrees counterclockwise about the embryo long axis. Each point represents a single embryo. Embryos display a preference for counterclockwise rotation (p = 0.001), but magnitude of rotation varies. **D,E**) Example of rotational correction between adjacent time points. **D,E**) Overlay of two *unc-130*::GFP nuclear segmentation channels (light, dark blue) from successive time points, before (**D**), and after (**E**) correction.

During morphogenesis, however, bulk embryo movements take place, such that the entire embryo rotates around the long axis (Fig 4; S1 Movie). Thus, the P2 and P3 axes are no longer time invariant. Tracking cell movement then becomes more complicated, because cell positions with respect to the fixed coordinate system of the early embryo are now governed by the composition of two transformations: one reflecting movement within a stationary P123 coordinate system, and the other reflecting bulk embryo movement. Because bulk movements vary widely in magnitude and even direction between embryos (average rotational correction of 34.8 +/- 47.5 degrees (mean +/- SD) counterclockwise, see below; Fig 4A–4C), automated alignments using a fixed coordinate system become impossible.

We therefore developed a method to computationally cancel embryonic rotations around the long axis, using the *unc-130*p::NLS::GFP fiducial marker. Although this reporter exhibits positional variability in its expression and additional variability in segmentation, we reasoned that neighboring time points of the same embryo likely exhibit only minor differences in expression and segmentation (Fig 4D and 4E), thus any differences seen would likely be attributable to bulk embryo movement, and could be corrected by back rotations along the long axis. This assumption was born out in subsequent calculations (Fig 4D).

We extracted 5000 *unc-130*::GFP points (voxels) at random from the segmentation data at a given time point for a given embryo and projected these onto the plane perpendicular to the long axis of the embryo. A similar operation was carried out for the subsequent time point. The resulting point clouds were fed to the point-matching program CPD-register [26–30], which generates rotations and translations to bring point clouds into correspondence (Fig 4D and 4E; Methods). Across all pairs of neighboring time points, rotations of 1.7 +/- 1.4 degrees (mean +/- SD) are observed. While implementing this procedure, we noticed that *unc-130*p::NLS::GFP nuclei exhibit small concerted linear movements independent of bulk embryo rotations, resulting in alignment artifacts causing the stationary eggshell, and the embryo inside it, to move with respect to the image frame through successive time points. We therefore computationally adjusted the transformations generated by CPD-register by applying a translational correction keeping all image-frame centers at the same position with respect to the camera, thus producing a pure rotation about the long axis in the P2-P3 plane.

With neighboring time points rotationally aligned, all image stacks could now be brought into correspondence and displayed on the same coordinate system as the first stack by summing or subtracting the individual rotations (S1 and S2 Movies). The coordinate system in the reference embryo was used as a reference set of axes onto which all other embryos were subsequently mapped (see below).

### Mapping test embryos onto the reference embryo coordinate system

Given identically-defined time-invariant coordinate systems for all embryos, alignment of any embryo to the reference requires only alignment of the coordinate axes. Having previously aligned the P1 axes, we calculated the rotation about this axis bringing the P2 axis (and therefore the P3 axis) into correspondence as described above (Fig 3F and 3G). This simple approach brought embryos substantially into alignment; however, inspection by eye suggested that P2 vector placement exhibited some variability from embryo to embryo. We therefore refined the alignment by matching point clouds from the rotated test and reference embryos at an early time point during the fast imaging period, using the CPD-register program as above (Fig 3H and 3I). The resulting dataset shows that on average a correction of 12.5 +/- 11.3 degrees produces a best fit.

Importantly, applying CPD-register without the initial coordinate axis alignment often gave very poor alignments, off by >60 degrees of rotation, suggesting that a number of local minimum solutions must exist, and that the correct one is obtained only if the embryos are already nearly aligned.

Since the P123 coordinate systems for each embryo were now aligned and time invariant, embryos were, in principle, registered over their entire time courses. Nonetheless, inspection of the alignments by eye again suggested that additional small improvements were possible, particularly near the onset of embryo twitching. Therefore, as a final correction, alignments at each time point were refined using CPD-register, with the source code altered to allow translations of arbitrary size but only small rotational corrections. The average angular correction introduced by this last step across all time points was 3.9 +/- 1.6 degrees (mean +/- SD), while the translational correction is 2.9 +/- 2.0 μm.

**Fig 5:**
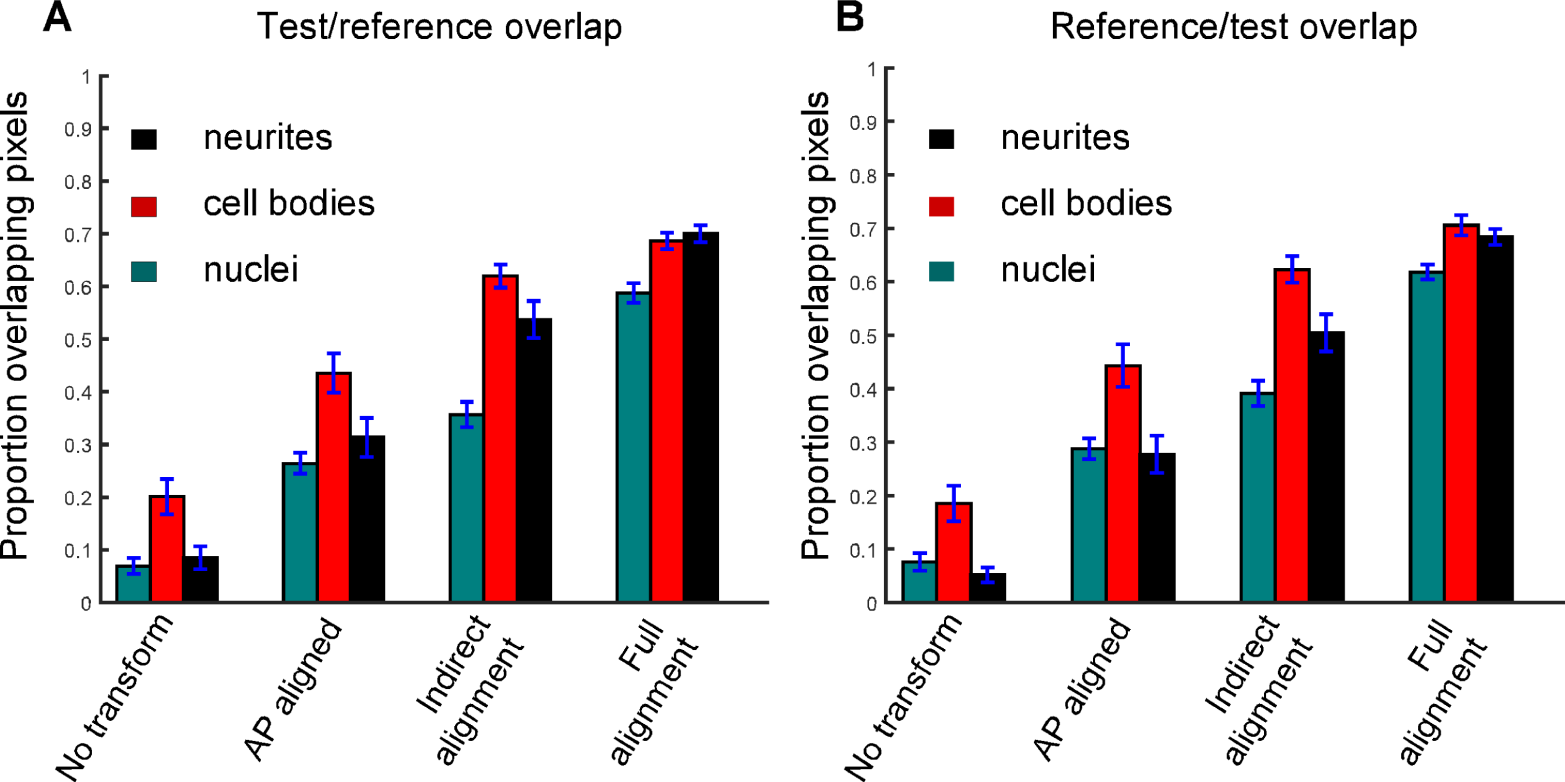
Quantitative measures of alignment accuracy. Quantitative measure of overlap between test and reference embryos, The proportion given is labeled pixels in the test/reference stack which are within 1 micrometer of a labeled pixel in the reference/test (**A, B**), respectively, averaged across test embryos. Image stacks are compared to reference at the last time point that could be matched prior to twitching. Overlap is calculated for four states: no transformation; long axis (AP) aligned; indirect alignment, all alignment steps except for final 3D refinement; full alignment, 3D refinement added. Error bars are S.E.M., calculated across embryos.

**Fig 6:**
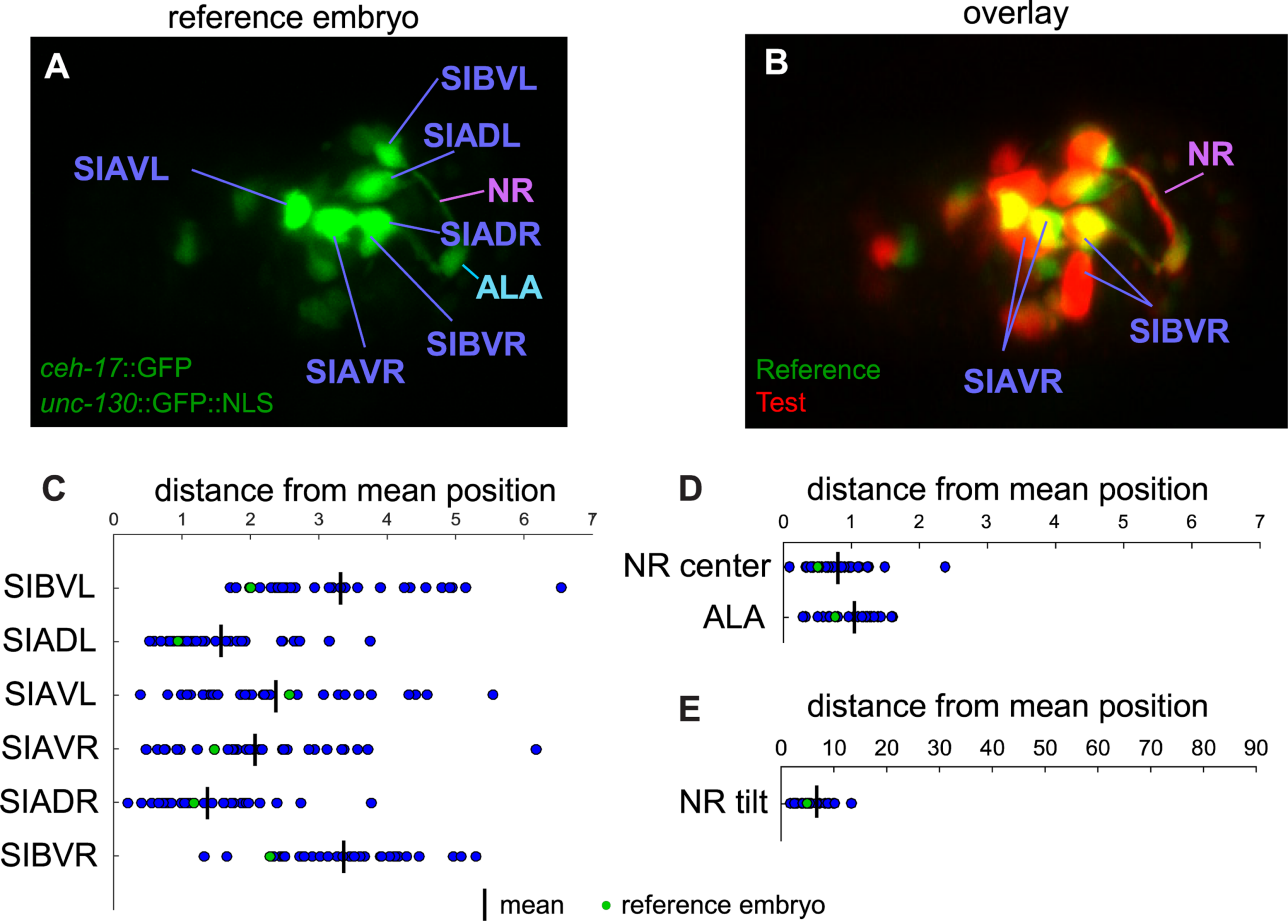
Quantitative measurements of wild-type nerve ring and cell body positions. **A**) Sublateral and ALA cell bodies in the reference embryo. NR, nerve ring. **B**) Overlap of a test embryo with the reference. Cell bodies in test embryos are identified with corresponding cell bodies in reference based on overlap at earlier time points in the fully aligned 4D sequences. **C**) Individual sublateral neuron cell body centers are measured by hand (Methods) at the last time point before twitching, and absolute value of distance from the mean position is reported. **D**) Nerve rings, NR, were traced out with a semi-automatic method in FIJI (Methods) and their centers measured by averaging over the traces, while ALA neuron center positions were measured by hand as with the sublateral neurons. Reported are deviations from the mean. **E**) The angle of the vector through the nerve ring was measured by fitting the nerve ring to a plane with a linear cost function. Deviations from the mean are reported.

This completed the full alignment procedure. Across our dataset, the algorithm produced excellent alignment by eye to the reference embryo in 30/33 of the test embryos. The three embryos scored as alignment failures demonstrated gross misalignment at early time points of the fast imaging period (~40 degrees of rotational error), resulting in one case from the initial definition of P2, and in the other two cases from the refining alignment at the early time points finding an incorrect minimum (perhaps facilitated by initial inaccuracy in definition of P2). To quantitatively follow the efficacy of our algorithm, we tracked overlap of the three segmented object classes between each test embryo and the reference as the alignment proceeds. As shown in Fig 5, each step results in a significant improvement in alignment. The maximal overlap of ~70% (and not 100%) is unlikely to reflect the need for better alignment procedures, as inspection of alignments by eye suggests that the fits are good. Rather, differences in segmentation details between embryos, and actual differences in cell locations (see below), probably account for the lack of full overlap. Typical alignments are shown in S3 Movie and in Fig 6A and 6B.

The procedure takes approximately an hour to run for a sequence of 71 351x251x288 image stacks on a 2.4 gHz Intel Xeon e5-2620 processor. Faster operation is possible if optimization thresholds are reduced in the CPD software. The software is adaptable to different stack sizes and/or number of stacks. Some of the steps can be optionally turned on/off. For example, the temporal alignment and the last refinement step are not always essential. It is also not always necessary to register each image to the subsequent one, and the number of skipped images can be adjusted. New StarryNite and Ilastik classifiers, however, do need to be generated for different gene reporter sets before running the alignment tool.

### Nerve ring, but not sublateral neuron cell body placement is precise

Having generated a dataset of 30 aligned embryos, we sought to determine the fidelity of NR placement in these embryos. We manually traced NRs using the *ceh-17*::GFP SubL neuron reporter in the final aligned time points before twitching using the Trak-EM2 extension to FIJI [31] (Methods). The NR centroid was calculated by averaging over the trace. We found that the aligned NR centers coincide to within 0.8 +/- 0.4 μm (mean +/- SD; Fig 6D). We also fit each NR trace to a plane using a linear fitting method (Methods), which would not give undue weight to non-planar sections near SubL neuron cell bodies. The measured planar angles agreed to within 6.0 +/- 2.9 degrees (mean +/- SD; Fig 6E). The ALA neuron is also labeled by the *ceh-17*::GFP reporter, and sits at the dorsal apex of the presumptive NR. Like the NR, the ALA cell body is also precisely positioned to within 1.0 +/- 0.4 μm (mean +/- SD; Fig 6D). The correspondence of ALA neuron cell bodies and SubL processes among embryos speaks to the precision of our embryo alignment algorithm as well as to the precision of the developmental events that generate these structures.

Surprisingly, unlike the SubL processes and ALA, SubL neuron cell body positions exhibit extensive variability (Fig 6C; Methods), with individual SubL cell bodies deviating by up to 6.5 um from the mean position of that cell body across all embryos (Fig 6C). While positional variation could stem from imprecise temporal alignment or natural variation in developmental clocks between embryos, this seems unlikely, given the good correspondence of *unc-130* hypodermal nuclei, ALA neuron, and the nerve rings in all three dimensions. Thus, while NR position with respect to the embryo body plan is highly precise, global positions of SubL cell bodies appear not to be tightly regulated (e.g. Fig 6A and 6B).

### *cam-1*/ROR is required for nerve ring position maintenance

Mutations in *cam-1*, encoding the *C*. *elegans* homolog of the ROR Wnt receptor, promote a small but significant anterior displacement of the NR in ~75% of larvae [17]. We wondered whether our alignment algorithm could be used to determine whether this defect results from early mispositioning of SubL pioneer neurons.

**Fig 7:**
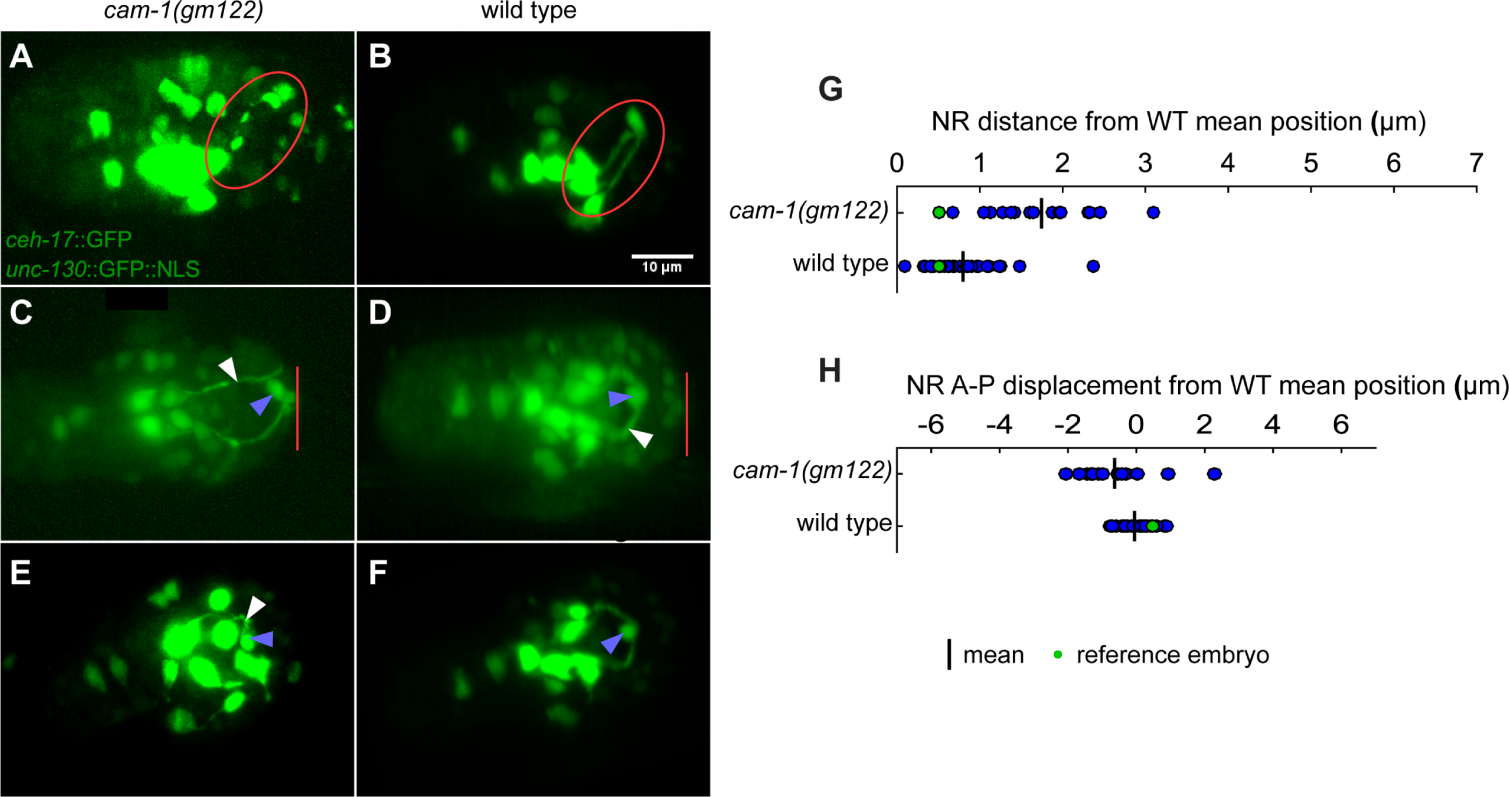
*cam-1* mutant defects and nerve ring alignments. *cam-1(gm-122)* mutant nerve ring defects (**A,C,E**) compared to wild-type embryos at similar time points, and which are rotated for comparison (**B,D,F**). White arrows, nerve ring. Blue arrows, ALA neuron. Red loops, nerve ring. Red bars, anterior end of embryo. **A,B**) Split nerve ring defect. The *cam-1* mutant animal arrested immediately after the nerve ring broke apart. **C,D**) Giant nerve ring defect. **E,F**) Disorganized nerve ring defect. **G,H**) phenotypically normal *cam-1(gm-122)* animals aligned to wild-type reference embryo and nerve ring deviations calculated as in Fig 6.

To do so, we imaged 39 *cam-1* mutant embryos using the same markers and imaging protocol described for the wild-type dataset above (Fig 7). Even before alignment, we observed a number of previously-unreported defects. One embryo exhibited highly disorganized SubL processes and cell body placement (Fig 7E and 7F), another exhibited severing of SubL processes entering the nerve ring (Fig 7A and 7B), while a third underwent non-specific arrest. None of these three embryos exhibited muscle twitching. 7/39 embryos exhibited a giant NR with a properly positioned posterior aspect, but an anterior apex displaced towards the tip of the nose (Fig 7C and 7D). In these animals, the ALA neuron also displayed inappropriate anterior placement (Fig 7C and 7D). To determine whether prolonged imaging might have perturbed the development of these animals, we imaged an additional 39 embryos starting shortly before NR closure with reduced light intensity relative to our other experiments (Table 1). Six of these animals had giant NRs, and one had disorganized SubL processes. We wondered whether the giant NR defect could explain NR anterior displacement in *cam-1* mutant larvae [17]. To this end, 30 of the 39 late-imaged animals were followed to hatching, including all embryos with a giant NR. 5/6 of giant NR animals failed to hatch, while among the phenotypically normal animals, only 2/22 failed to hatch. Thus, the giant NR defect, occurring relatively rarely in embryos and more rarely still among successfully hatched animals, is unlikely to explain the anterior NR displacement observed in most *cam-1* mutant larvae.

**Table 1:** Hatching rates of *cam-1(gm122)* animals.

Phenotype	No. embryos	No. hatching	% hatching
Normal NR	24	22	92
Large NR	6	1	17

NR, nerve ring. The low hatching rate and low incidence of animals with a large nerve ring suggests they may not be the animals exhibiting anterior nerve ring defects reported in [11].

Based on these observations, we proceeded to align 20 embryos that exhibited twitching and that did not contain a giant NR to the wild-type reference embryo (S3 Fig). Two of these showed very poor alignment, likely because expression of the *unc-130*p::NLS::GFP reporter appeared altered, and thus, NR positions could not be quantitatively assessed. The remaining 18 animals appeared by eye to align, although in some cases the last step of the alignment protocol had to be omitted, suggestive of increased variability in *unc-130*::GFP nuclei placement at later, but not earlier, stages. Two of the aligned animals exhibited gross asymmetry in left-right outgrowth rates of SubL neuron bundles. Quantitative assessment of NR position and angle with respect to the reference embryo in the remaining 16 embryos revealed no obvious anterior displacement of the NR (Fig 7G and 7H), although overall positioning seemed to be somewhat more variable in *cam-1* animals than in wild-type animals.

Taken together, these observations suggest that the anterior NR defect observed in *cam-1* mutant larvae is unlikely a result of early NR misplacement, and may reflect a maintenance role for the gene. In this respect, CAM-1/ROR function appears to be similar to that of SAX-3/ROBO, mutations in which also promote maintenance-related anterior NR placement defects [32].

## Discussion

The acquisition of quantitative data on the fidelity and reproducibility of developmental processes requires the precise spatiotemporal alignment of a sufficient number of animals to make statistical claims. A number of obstacles, including developmental variability, imaging variability, and data processing variability make alignments non trivial. Here we describe an automated method for aligning 4D *C*. *elegans* embryonic imaging data between the 50–100 cell stage and the onset of muscle twitching. We demonstrate that progressive steps in the alignment procedure result in increasing overlap of signals from fiducial reporters used for alignment. Perhaps more relevant, the precision with which nerve rings are aligned by the algorithm suggests that our approach produces results accurate to within less than a micrometer, although absolute error is likely greater for features closer to the lateral surfaces of the embryo, given the importance of rotations about the long axis of the embryo to the alignment algorithm. Key to efficient alignment is our observation that applying global point-matching algorithms without initially bringing embryos into rough correspondence often fails. This reflects a tension between competing needs: using enough fiducial points to combat variability in their segmentation (due to imaging orientation, signal variability, etc.), and using sufficiently few fiducial markers to limit the number of configurations that provide maximal signal overlap. For this reason, and because of the underlying variability in segmentation, expression, etc., as well as the presence of partial symmetries in expression patterns (resulting in local maxima), an overlap parameter does not adequately capture the success of an alignment. Future refinement of alignment methods may, therefore, focus on the development of more complex parameters, accounting not only for raw signal overlap, but also taking into account subtler features of the aligned images. Improvements to our work may also involve extending alignments beyond twitching onset. This problem is computationally challenging as the animal changes its configuration in non-rigid ways, requiring more frequent imaging and the development of non-rigid alignment schemes. Recent semi-automated segmentation approaches may offer an excellent starting point [15].

Our alignment procedure allowed us to consider a number of questions related to the fidelity of *C*. *elegans* embryonic development. We demonstrated that positioning of the NR brain neuropil in the embryo is accurate to within about a micrometer. This result is all the more impressive as some of the variance we observe must be due to errors stemming from limited optical resolution, imprecision in the alignments, and measurements of neurite positioning. It is therefore likely that NR placement is accomplished with an accuracy of much less than a micrometer. Conversely, we showed that SubL neuron cell body placement can vary up to six micrometers between individuals. As the head is ~25 micrometers in diameter, this reflects ~25% relative variability. This result strongly contrasts with the prevailing notion that the fixed lineage of *C*. *elegans* should lead to fixed cell placement [2,33]. It also suggests that strong selective pressures on NR placement exist. Consistent with this idea, our alignments reveal that the onset of muscle twitching in the embryo is temporally precise, and occurs within a defined developmental interval of <7 minutes. This represents a variation of ~6% as embryos are synchronized using our temporal alignment protocol through an interval 116 minutes, on average, before twitching. Twitching is correlated with dorsal arrival and overlap of left-right SubL neuron axon bundles, suggesting that precision in SubL neuron axon placement may promote temporal precision in muscle activation.

Our results raise the possibility that the ALA neuron may play a role in defining the dorsal meeting point of the left and right SubL bundles. ALA is located on the dorsal midline and appears to be within the optical resolution limit (~1 micron) of SubL processes, roughly at the point at which the two arms of the nerve ring cross. Unlike SubL neuron cell bodies, the ALA cell body exhibits <1 micron variability in placement between embryos. Furthermore, in *cam-1* mutant animals exhibiting giant NRs, ALA is displaced anteriorly, but is located at the same relative position with respect to the NR. Thus, ALA could define a guidepost towards which NR pioneers grow.

Finally, although the method we present here relies on the specific distribution of *unc-130*p::NLS::GFP expression, used to generate fiducial points for alignments, other reporters expressed in a similar number of different cells could be used as well. In this case, the specifics of the initial rough orientation of the embryo would be different, but subsequent refined alignments would still follow the same algorithms presented here. Importantly, however, some asymmetry in reporter expression would be important to allow precise definition of axes perpendicular to the long embryonic axis. The *ceh-17*::GFP reporter is not used in alignment, and a reporter expressed in any other system of the embryo could be used in its place. Thus, with relatively small manipulations, the method we present here can be applied to other reporter combinations in *C*. *elegans*, perhaps permitting mapping of a variety of tissues in the animal prior to twitching.

Extension of our alignment scheme to other model systems would require identification of pan-nuclear markers and an asymmetric fiduciary reporter. As is the case for post-twitching *C*. *elegans*, our method is likely not suited for registering image stack series for which local transformations, and not a global affine transformation, represent the geometry.

## Methods

### Strains

*C*. *elegans* strains were maintained at 20°C under standard conditions [34]. The OS10584 strain, which is the wild-type strain, contains three transgenes: *stIs10116* (*his-*72 promoter::*his-24*::mCherry::*let-858* 3'UTR + *unc-119*(+)); *wgIs76* (*unc-130* promoter::TY1::eGFP::3xFLAG(P000007_E01)); and *npIs1* (*ceh-17* promoter::GFP, pRF4). It was generated by crossing the RW11144 strain (obtained from the *Caenorhabditis* Genetics Consortium) into the IB8 strain [35]. The OS10585 strain was generated by crossing the OS10584 strain into the NG2615 strain containing the *cam-1(gm122)* mutation [16]. The *his-72* promoter labels most cells of the embryo, with weak to no expression in the germ line, depending on stage, and weak expression in intestine [36].

### Microscope design

All images were taken on a custom-built single plane illumination microscope based on the design of Wu et al. [15]. Briefly, light is collected from a 488nm laser and a 588nm laser and combined using a beamsplitter (Thorlabs). Color switching is accomplished using an acoustic optical tunable filter (AOTF) from AA-Optoelectronics to turn excitation channels on and off, with both green and red light ultimately collected through a Chroma dual color filter (59022m). Lasers are scanned in two orthogonal directions using 2 1D galvanometers (Thorlabs) optically conjugate to the sample to generate the light sheet and volume. Lasers are focused onto the sample through a 40x 0.8 NA Nikon water-dipping objective, and emission light is collected from an identical 40x objective. A third objective (10x 0.3 NA, Olympus) is used for sample finding. The microscope body, which holds the objectives in position and provides control over the piezo and the sample position, is provided by ASI. The emission objective tracks the light sheet using a piezo-electric element purchased from ASI. All images were taken using a Hamamatsu Orca Flash 4.0 v.2 sCMOS camera. Control software was custom written by us in Labview. Electronic elements were synchronized using a mix of software synchronization provided by Labview and TTL voltage synchronization through a pair of NI PCIe 6363 DAQ cards. The microscope design and optical path is shown in S4 and S5 Figs.

### Microscope operation

All image stacks were acquired with both green and red channels, using a 488 nm and a 588 nm laser respectively for excitation. Laser power was measured just in front of the 500 mm lens. 488 nm laser power was measured at 165 +/- 5 μW, and the 588 nm laser at 120 +/- 3 μW, except for the late imaging experiments in *cam-1* (see below). The laser was scanned back and forth once for each imaging slice to prevent directional effects which were otherwise observed in the imaging. A 20 ms delay was provided between every slice to give time for the piezo to settle at its final position, 10 ms was provided for the actual imaging, a ~3 ms delay was used giving the rolling shutter on the CMOS camera time to complete its readout, and 2 ms additional used for software synchronization, resulting in 35 ms total per slice. All stacks consisted of 100 slices, and both red and green channels were acquired for all stacks, so each stack took 7 seconds in total. Stacks were subsequently truncated to 93 slices to remove occasional bad slices at the top and bottom of the volume and to overlay the red and green channels, which were slightly offset in Z. Up to 6 embryos could be imaged at once given the timing considerations outlined above. Embryos were kept well separated to prevent the excitation light targeting one embryo from affecting another.

For most visualization, registration, and position calculations, stacks were linearly interpolated in Z to produce uniform voxels. Stacks were also trimmed in *x* and *y* to more closely match the size of the embryos. The final stacks were 351 x 251 x 287 voxels, with voxel size being 162.5 nm x 162.5 nm x 162.5 nm, for an overall frame size of 57.0 μm x 40.8 μm x 46.7 μm.

For the late *cam-1* imaging experiment, a lower power of ~120 μW in 488 nm and 100 uW in 588 nm were used–the mCherry channel was also acquired in addition to GFP for a possible use in alignment and visualization (although not used). These lower imaging powers and late onset of imaging eased concerns that photodamage might contribute to the large NR defect previously observed, or prevent animals from hatching.

## Sample preparation

24 mm x 50 mm coverslips (Fisher) were spotted in the center with 30 μl of .01% poly-l-lysine solution (Sigma), let sit until dry, then stored at least a day before use to provide time for the polylysine to firmly adhere to the coverslips. Only coverslips for which the dry polylysine pattern overlapped the exact middle were used, as the imageable space on the coverslip given the constraints of the SPIM system is only the central ~6 mm x ~3 mm.

Embryos were collected for imaging by cutting gravid hermaphrodites in their first 36 hours of laying, in ddH2O. Embryos were mounted in ddH2O on the polylysine coated glass using a mouth-pipette apparatus (Fisher) and a drawn 1 mm capillary with the end broken off. Embryos were blown out of the capillary into an acceptable (central) *x-y* position before beginning. As embryos fell, they were gently rotated into approximately the optimal orientation, flat on the coverslip and perpendicular to the excitation axis, by blowing fluid on them or sucking fluid into the capillary as they fell. When embryos reached the coverslip they rapidly adhered and could no longer be manipulated. The desired orientation, perpendicular to the beam, minimizes expansion of the laser across the width of the embryo, thus leading to slight improvements in sectioning and light efficiency. However, as the placement procedure is difficult to implement and many embryos could not be brought into exactly the correct orientation, embryos were accepted for imaging that fell within 45 degrees of the correct angle (i.e., two octants' worth, or 1/4 of embryos if they had fallen in random orientations). Once the embryos were stuck, the coverslips were washed 3–5 times in water to remove all loose polylysine, in order to protect the water-dipping SPIM objectives.

After all embryos were mounted, the time was noted and embryos were chosen for imaging that were between the 1 and 6 cell stages (which staged them to within about an hour). The embryos were subsequently let sit for 2 hours at 20°C before start of imaging. All imaging was carried out in a 20°C temperature controlled room.

After waiting two hours, we imaged the embryos every 10 minutes for 20 stacks (T1-T20), followed by faster imaging every 2 minutes for 125 stacks (T21-T145) which covered the period leading from before NR development through twitching. The resulting embryos were positioned at an arbitrary dorsal-ventral and left-right position about their long axis since they are uncompressed and so fall into arbitrary rotational orientations (unlike [2]).

For the reference, we chose an embryo initially positioned with its long axis very nearly perpendicular to the detection objective axis, with an initial rotational positioning about its long axis such that NR outgrowth was easily visible. This embryo had been observed to hatch at a later time point, so that it was known to be healthy (this data was only available for some of our embryos).

In the case of OS10584, 46 embryos were initially collected over 7 days worth of imaging. Subsequently, 2 days worth (12 embryos) were discarded, since they featured substantial contamination on the coverslips, which caused incorrect mCherry channel segmentation (see below) on several of the embryos. 33 embryos were set up for alignment (not including the reference, which made 34). Of these, 30 embryos looked acceptably aligned by eye while 3 exhibited obvious misalignments (~40 degree rotations) stemming from misrotations at the early time point.

For the OS10585 imaging, conducted in the style of OS10584, 39 animals were set up for imaging. We attempted alignments on the apparently normal animals, of which many were discarded before conducting alignments for various reasons, including absence of twitching (precluding temporally matching the embryos at their twitching times), bacterial contamination of the coverslips resulting in failure of mCherry channel (StarryNite) segmentation, or movement of the stage during imaging (over one day, five embryos). For the late-imaged *cam-1* animals, an additional 39 animals were imaged starting before the closure of the NR, giving at least 20 hours from fertilization for hatching to occur (compared with the 14 hours typical in wild type).

### Image analysis

Red channel nuclear segmentation was done using the StarryNite program [23]. We made very slight modifications to the program for our use, affecting only superficial aspects of the program's data handling and interface. StarryNite parameters for wild-type (OS10584) experiments were determined manually by trial and error, working on an RW11144 dataset with imaging conditions identical to those described for OS10584. The parameters were adjusted for the *cam-1* embryos, OS10585, to improve segmentation efficiency. As *cam-1* temporal alignments often yielded bad results (with our program finding a degenerate solution in some cases, and large offsets at the last time point in others), *cam-1* animals were not aligned temporally using the StarryNite data, but rather aligned using the last time point before twitching as a guide.

A multiple of the red channel signal intensity (determined at 1/15 for these imaging conditions) was subtracted from the green channel before analysis, to eliminate bleedthrough of the red channel, which was otherwise substantial.

Green channel segmentation of cell bodies, neurites, and nuclei used Ilastik [24], a random forest [37] machine learning method that segments images based on a combination of classifiers such as raw intensity, Difference of Gaussians, and Laplacian of Gaussian. Segmentation of *unc-130*p::NLS::GFP nuclei for determining the anterior end of the embryo, for estimating the initial DV-LR angle, and for correcting the initial estimate of DV-LR angle were done simply by thresholding the (0 1) probabilities output by Ilastik to produce a binary matrix. The threshold criterion was relaxed slightly for segmentation procedures in which more than the usual number of nuclei were desired (i.e., early time point DV-LR angle estimation, determination of the anterior end of the embryo, and determination of nuclei in the target embryo for the refinement step of the algorithm). For 2D rotational corrections within a given embryo and 3D alignments between embryos, *unc-130*p::NLS::GFP segmentation was improved by coring out the area around the NR using a cylinder of defined size, blurring with a Gaussian filter, and restricting the segmentation results to the largest visible objects (maximum of 17 objects).

Temporal alignment of embryos was carried out by minimizing square error over possible offsets of a test embryo relative to the first 20 time points of the reference, normalized by the number of overlapping time points in the solution–i.e., if a test embryo is offset by 5 time points, 15 time points continue to overlap with the first 20 in the reference. Without this normalization, offsetting a test embryo by the maximum number of time points usually minimizes the square error. Nuclear number time courses were interpolated by a factor of 5 prior to alignment as described in the main text. For finding plateaus, all time points were collected for which the number of segmented nuclei was between 120 and 350, then divided into two sets for the two plateaus, 120 to 220 and 220 to 350. A subset of these time points was then defined for which the absolute value of percentage change of (interpolated) cell number from the previous time point was less than or equal to a defined threshold (initially, 0%). The 0% threshold was increased by small increments until a connected component of more than fifteen time points was found, which was selected as a plateau; the two plateaus found in this way for the two groups correspond to the 200 cell stage and the ball stage of embryogenesis. The time associated with each plateau was defined as the mean of the time points in the connected component. Correlation analysis was then conducted with twitching time as described in the text.

Embryo scaling factors were treated symmetrically between the short axes of the embryo, because the second and third moments are similar in magnitude and the associated axes vary in time. The average ratio between short axis moments over the period considered was 1.04 +/- .03 (mean +/- SD), and it was highly correlated across embryos (r = 0.96). In addition, the direction associated with these moments varied over repeated calculations for each embryo by an average of 8.9 +/- 7.7 degrees (mean +/- SD) across time points in all embryos. For comparison, using the same calculation, random reorientation at each step would give 45 degrees deviation). By contrast, the long-axis moments deviate from the other two more substantially, by a factor of 2.05 +/- .10 (mean +/- SD) across embryos, are less well correlated with the other two (r = 0.81), and their angles deviate much less over repeated measurements (1.2 +/- 2.0 degrees deviation per embryo; mean +/- SD across embryos). Given the limited and variable separation of the short axis moments from one another, we ultimately calculated only two scale factors for length and width, averaging the results for width over the short axes.

Embryo scaling factors were calculated as follows. For a given embryo, let *a* be the moment about the long axis, and *b***,** the average moment about the short axes, with the subscripts “*ref*” and “*test*” referring to the reference and individual test embryos, respectively. Let:

$$q_{1}=\frac{a_{ref}}{a_{test}}\;\mathrm{and}\;q_{2}=\left(b_{ref}-\frac{a_{ref}}{2}\right)/\left(b_{test}-\frac{a_{test}}{2}\right)$$

Then the ratios that must be applied to a test embryo to bring long and short axes into correspondence with the reference, *r*_*l*_ and *r*_*s*_ respectively, are given by:

$$r_{l}=q_{1}^{-\frac{1}{5}}*q_{2}^{\frac{2}{5}}\;\mathrm{and}\;r_{s}=q_{1}^{\frac{3}{10}}*q_{2}^{-\frac{1}{10}}$$

These relations can be easily verified by considering the moment of inertia integrals as integrals of a density over space, and considering the change in this density as a result of (uniform) local scaling. The reason for the somewhat exotic exponents appearing in the formula is that the moments of inertia scale as the 5^th^ power of distance (3 stemming from the mass density, 2 from the distance weighting).

Point cloud matching was carried out using the "cpd_rigid" alignment method of the Coherent Point Drift (CPD) registration program. CPD register is a Gaussian Mixture Model method using a deterministic annealing procedure to shrink the size of the Gaussians over time. We chose it from among other possibilities since it has a Matlab interface, which matched well with our programming environment, and because it seemed to give better results than tests with the older Iterative Closest Point Method. In addition, the theoretical underpinnings of the method seem to be stronger than some others, with exact instead of approximate solutions to the underlying mathematical model. For 3D alignments between embryos (the refinement step of the algorithm), CPD register was altered to bias against rotations by directly reducing the size of calculated rotations until a certain Gaussian search width had been reached, at which point full rotations were gradually reintroduced. The effect of this approach is to permit only rotations that match nuclei to nearby nuclei.

SubL and ALA cell body center positions were calculated manually in FIJI [31] with center positions in *x* and *y* estimated by eye from maximum projections and *z* positions found by scrolling through stacks at the central *x-y* position until the slice with maximum brightness at that position was found. When the max projection *x-y* center was seen to be substantially off-center at the maximum brightness slice, the *x-y* center was recalculated based on apparent center positions at that slice (resulting in corrections typically of up to a few hundred nanometers). Identification of cell bodies in test embryos with cells in the reference is reliable and based on previous colocalization at earlier stages of embryogenesis (using the full aligned movies). Subsequent identification with particular sublateral cell bodies and the ALA is accomplished by comparing the descriptions of the *ceh-17* marker provided in [35] with the diagrams in [1]. The actual nomenclature is tentative and may be incomplete, as SIADL and SIADR sometimes appear to have another immediately neighboring fluorescent cell. This may be an artifact of the imaging or image transformations, or a visual misunderstanding of the underlying shapes of these two neurons. For SIADL and SIADR the apparent center position of the cluster was taken, with no obvious effect on the variability of the positioning.

Neurites were traced using the TrakEM2 add-on to Fiji, using the semi-automatic tracing method ("pencil") with short (~5–10 pixel) steps between automatic tracing runs. When the automatic tracing method appeared to deduce an incorrect trace, the step size was shortened until the neurite was correctly traced. Short stretches were occasionally manually added ("pen") when the pencil could not find the right route. The main benefit of using the semi-automatic functionality here was that it seemed much more accurate in tracing neurite movement between image slices in Z than could easily be done by hand. Tracing was done on the 93 slice stacks before interpolation to 287 slices, since determining the center slices of neurites was found to be somewhat easier here than using the interpolated version, and the TrakEM2 program ran better with smaller image stacks. Traces are exported as XML files, read in Matlab, and converted to arrays and matrices for further modification and analysis. NRs were fit to a plane by setting an approximate center line (based on observation of the reference embryo and assuming good alignment of other NRs to reference), dividing the nerve ring into top and bottom arms based on position relative to this center line, identifying vectors between points on the top and bottom arms of the ring and decomposing into two sets; a side-to-side vector, which is the component-by-component average of the vectors between points in the bottom and top arms of the plane; a forward vector, which is the average of resulting vectors after subtraction of components in the direction of the side-to-side vector; and an “out” vector, which is the perpendicular to the forward and side-to-side vectors, normalized in all cases to lie in the same half-sphere. This “out” vector defines the plane and is used in the calculation of angles between NRs described in the main text. This method was used instead of a least squares fit because square error seemed to overfit to non-planar segments of the ring near the sublateral neuron cell bodies.

Except for some of the external programs described above which were incorporated in our overall image registration pipeline, all image processing was done using custom-built code written in Matlab, and is available upon request.

## Supporting information

S1 Fig Overview of spatial alignment process. Green text, outcome of preceding steps.
(PDF)

S2 Fig Quantifying segmentation procedure accuracy. **A**) Additional measures of segmentation for cell bodies and neurites are compared to Ilastik segmentation using the “pixels within 1 micrometer” method described in the text. The neurite segmentation channel for the last time point is compared to manual traces. The cell body segmentation is compared to a simple filter that extracts all pixels above 500 counts. Proportion of labeled pixels in test image within 1 micrometer of a labeled pixel in reference, and vice versa, are shown. **B**) The false positive rate in nuclear segmentation was calculated by hand using 90 time points from random embryos, distributed throughout embryogenesis. 13 false positives were recorded out of 1213 nuclei counted by the full nuclear segmentation procedure. **C**) Nuclear counts per time point across all time points, recorded using the full segmentation procedure. The minimum count across all time points is 6 nuclei, while the maximum recorded (which is the maximum permitted by the software) is 17. Except in highly symmetrical cases, only 3 nuclei are required to match two 3D objects.
(PDF)

S3 Fig Alignments of phenotypically normal *cam-1* animals to wild type. Alignment procedure as described in the text, with slight modifications because of poor segmentation of nuclei in red and green channels. An embryo aligned to reference at the second to last time point (**A-C**), and at the last time point (**D-F**).
(PDF)

S4 Fig SPIM optical table light path. Diagram of external light path for the custom-built SPIM microscope used in the experiments described in the text. Briefly, 488 nm and a 588 nm laser light are combined with a long-pass beamsplitter, and directed to an acoustic optical tunable filter (AOTF) used for beam shuttering. The beam encounters a pair of galvanometers used to scan the beam and generate the light sheet and volume. Mirrors and relay lenses are used to reimage the galvanometers onto the back focal plane of the excitation objective, converting the angular shifting of the laser beams at the galvos into linear movement at the sample. **A**) Front view of microscope. **B**) The light path is traced on the optical table, with the periscope and back port of the microscope broken out for a clearer view (**C**). **D**) Diagram of the light path through the optical elements on the table. Mirror 1 is conjugate to the sample plane and can be used to adjust angles at the sample. Mirrors 2 were used in setting the optical distance to the periscope. **E**) Legend for **B**, **C**, **D**.
(PDF)

S5 Fig Microscope body light path. A diagram of the light path in the microscope body. Excitation light (purple) flows down through the excitation objective (“Ex”) to the sample, producing fluorescence (magenta), which is collected by the emission objective (“Em”) and conveyed to a Hamamatsu SCMOS camera. A second lens system and camera on the bottom axis, imaging at low NA, provides the capacity to locate samples for imaging. **A**) Front view of microscope. **B**) Overlay of light path on microscope body. **C**) Diagram of light path and optical components. **D**) Legend for **B**, **C**.
(PDF)

S1 Movie Development of the reference embryo. (AVI)

S2 Movie Reference embryo with rotations corrected. (AVI)

S3 Movie Test embryo aligned with reference embryo. (AVI)
