# Supplementary material for: Automated C. elegans embryo alignments reveal brain neuropil position invariance despite lax cell body placement
Source: PLoS One. 2018 Mar 28;13(3):e0194861. doi: 10.1371/journal.pone.0194861 (PMC5874040; doi:10.1371/journal.pone.0194861)
Supplement: S1 Fig — Green text, outcome of preceding steps. (PDF) [file pone.0194861.s001.pdf]

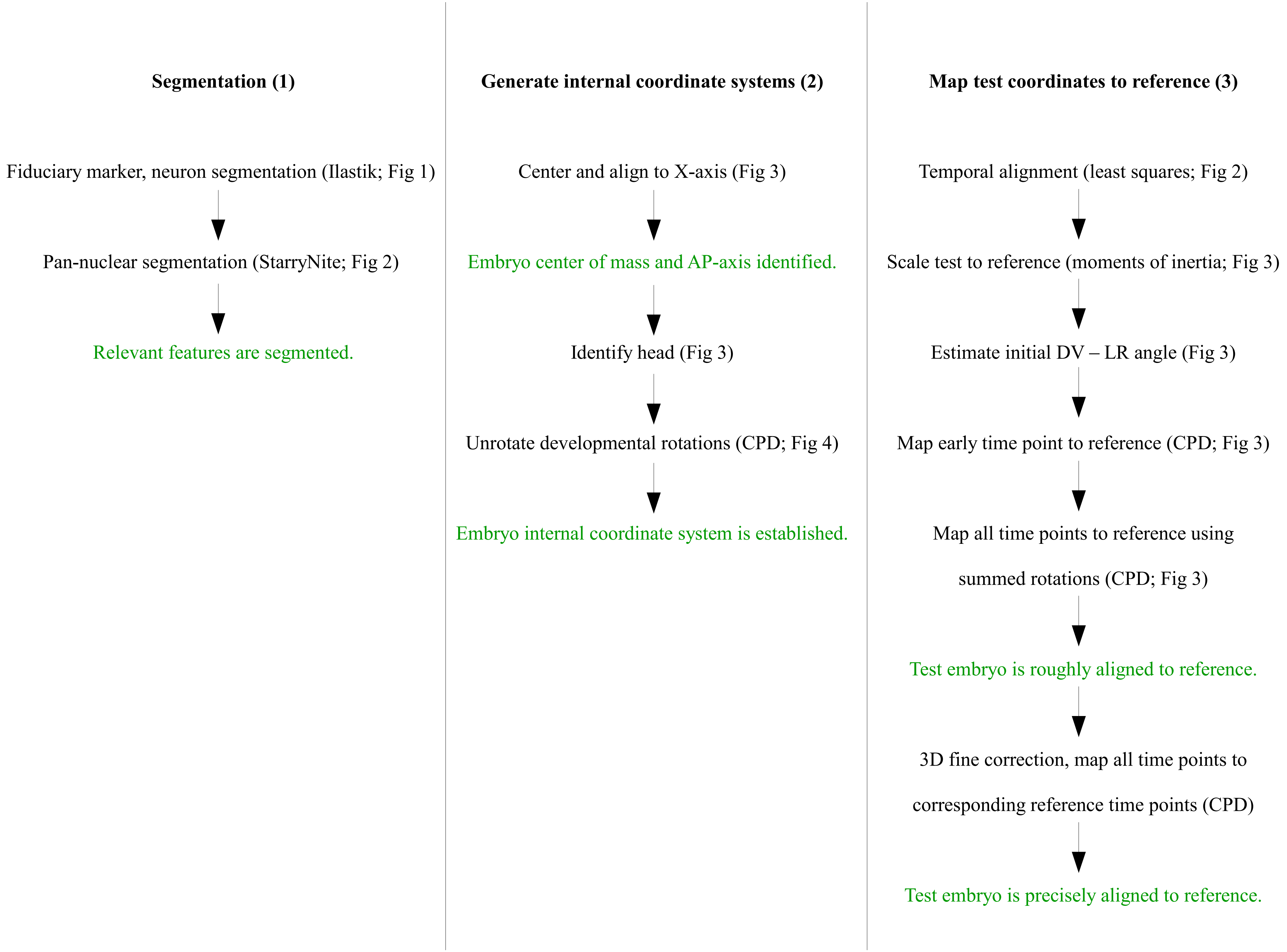
Segmentation (1)
Fiduciary marker, neuron segmentation (Ilastik; Fig 1)
Pan-nuclear segmentation (StarryNite; Fig 2)
Relevant features are segmented.
Generate internal coordinate systems (2)
Center and align to X-axis (Fig 3)
Embryo center of mass and AP-axis identified.
Identify head (Fig 3)
Unrotate developmental rotations (CPD; Fig 4)
Embryo internal coordinate system is established.
Map test coordinates to reference (3)
Temporal alignment (least squares; Fig 2)
Scale test to reference (moments of inertia; Fig 3)
Estimate initial DV – LR angle (Fig 3)
Map early time point to reference (CPD; Fig 3)
Map all time points to reference using summed rotations (CPD; Fig 3)
Test embryo is roughly aligned to reference.
3D fine correction, map all time points to corresponding reference time points (CPD)
Test embryo is precisely aligned to reference.
