# Supplementary figures and images for: Automated C. elegans embryo alignments reveal brain neuropil position invariance despite lax cell body placement
Source: PLoS One. 2018 Mar 28;13(3):e0194861. doi: 10.1371/journal.pone.0194861 (PMC5874040; doi:10.1371/journal.pone.0194861)

# Insley and Shaham, Figure S2

**A**

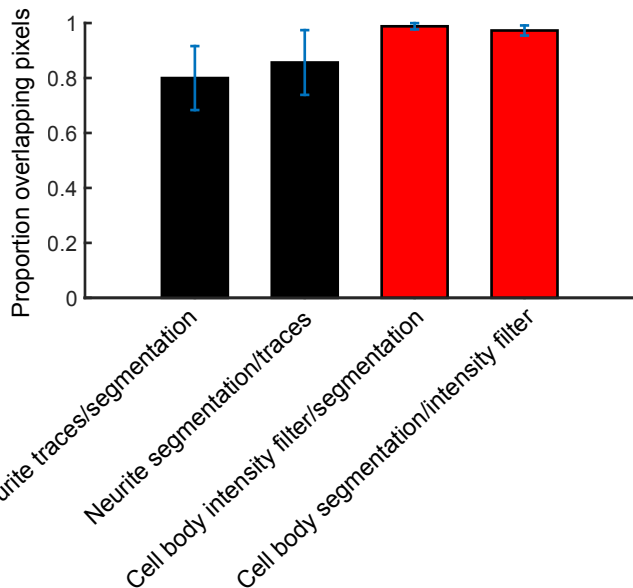

**B**

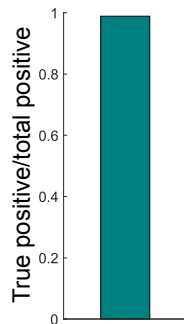

**C**

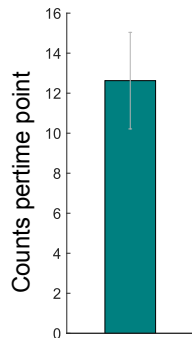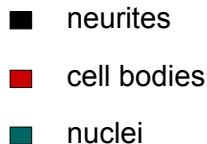

Supplement: S2 Fig — A) Additional measures of segmentation for cell bodies and neurites are compared to Ilastik segmentation using the “pixels within 1 micrometer” method described in the text. The neurite segmentation channel for the last time point is compared to manual traces. The cell body segmentation is compared to a simple filter that extracts all pixels above 500 counts. Proportion of labeled pixels in test image within 1 micrometer of a labeled pixel in reference, and vice versa, are shown. B) The false positive rate in nuclear segmentation was calculated by hand using 90 time points from random embryos, distributed throughout embryogenesis. 13 false positives were recorded out of 1213 nuclei counted by the full nuclear segmentation procedure. C) Nuclear counts per time point across all time points, recorded using the full segmentation procedure. The minimum count across all time points is 6 nuclei, while the maximum recorded (which is the maximum permitted by the software) is 17. Except in highly symmetrical cases, only 3 nuclei are required to match two 3D objects. (PDF) [file pone.0194861.s002.pdf]

# Insley and Shaham, Figure S3

embryo 1 overlap

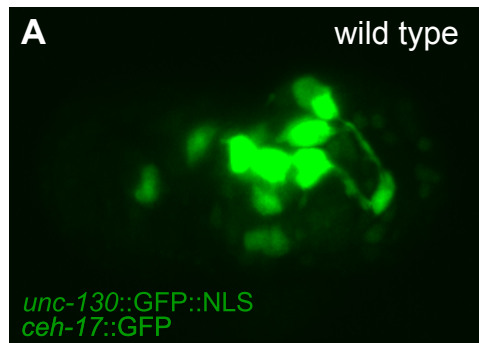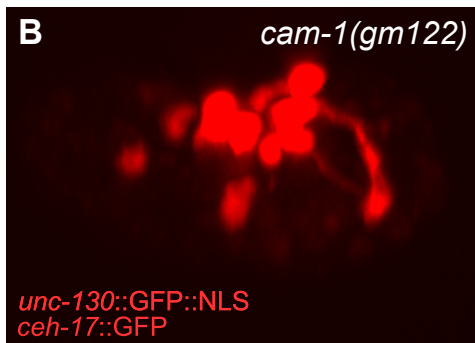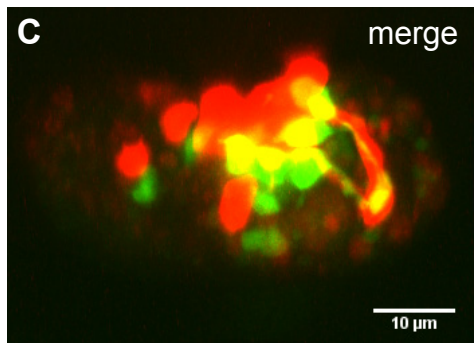

embryo 2 overlap

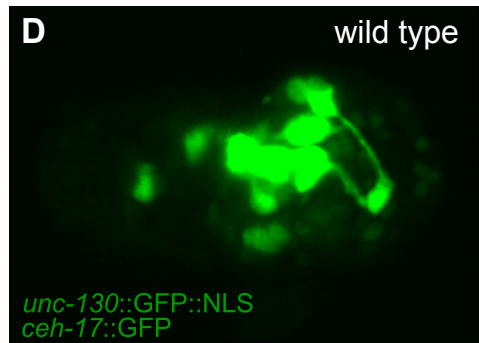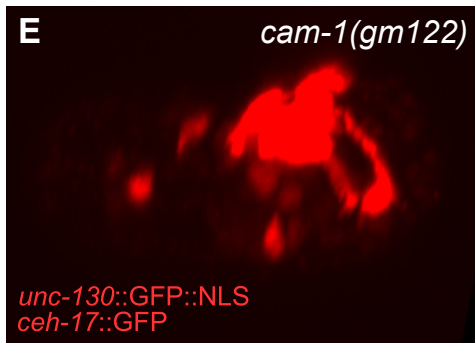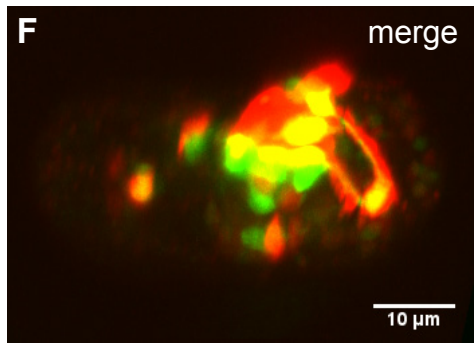

Supplement: S3 Fig — Alignment procedure as described in the text, with slight modifications because of poor segmentation of nuclei in red and green channels. An embryo aligned to reference at the second to last time point (A-C), and at the last time point (D-F). (PDF) [file pone.0194861.s003.pdf]

# Insley and Shaham Figure S4

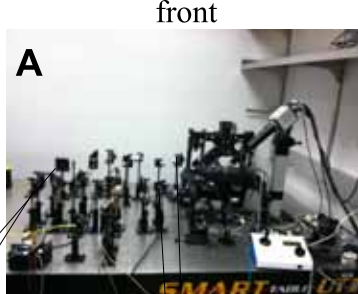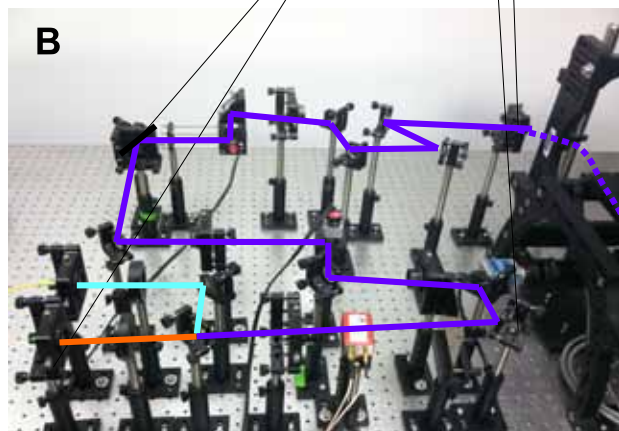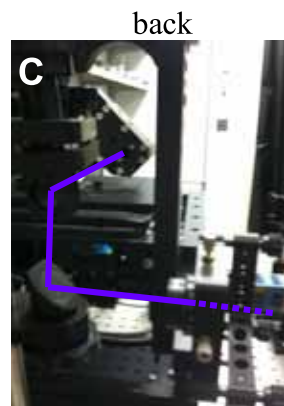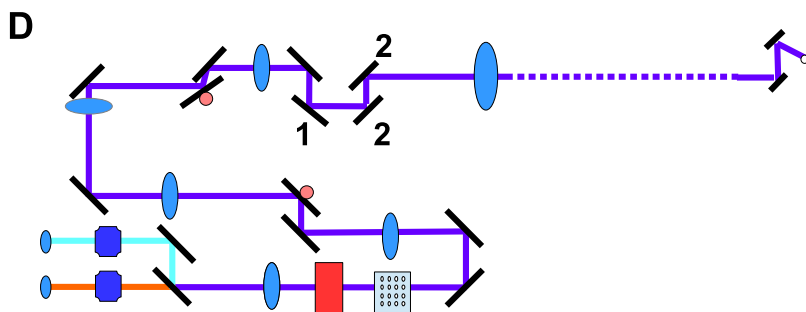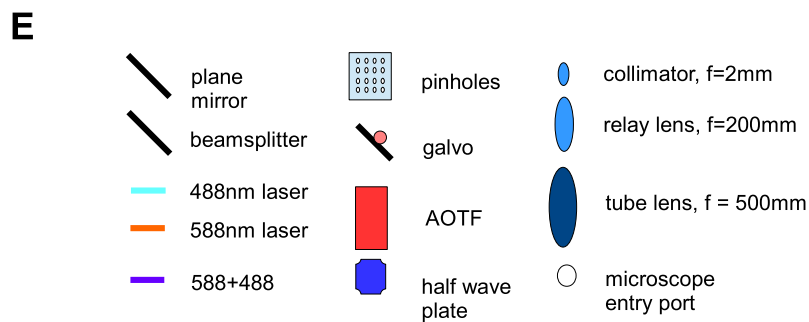

Supplement: S4 Fig — Diagram of external light path for the custom-built SPIM microscope used in the experiments described in the text. Briefly, 488 nm and a 588 nm laser light are combined with a long-pass beamsplitter, and directed to an acoustic optical tunable filter (AOTF) used for beam shuttering. The beam encounters a pair of galvanometers used to scan the beam and generate the light sheet and volume. Mirrors and relay lenses are used to reimage the galvanometers onto the back focal plane of the excitation objective, converting the angular shifting of the laser beams at the galvos into linear movement at the sample. A) Front view of microscope. B) The light path is traced on the optical table, with the periscope and back port of the microscope broken out for a clearer view (C). D) Diagram of the light path through the optical elements on the table. Mirror 1 is conjugate to the sample plane and can be used to adjust angles at the sample. Mirrors 2 were used in setting the optical distance to the periscope. E) Legend for B, C, D. (PDF) [file pone.0194861.s004.pdf]

# Insley and Shaham, Figure S5

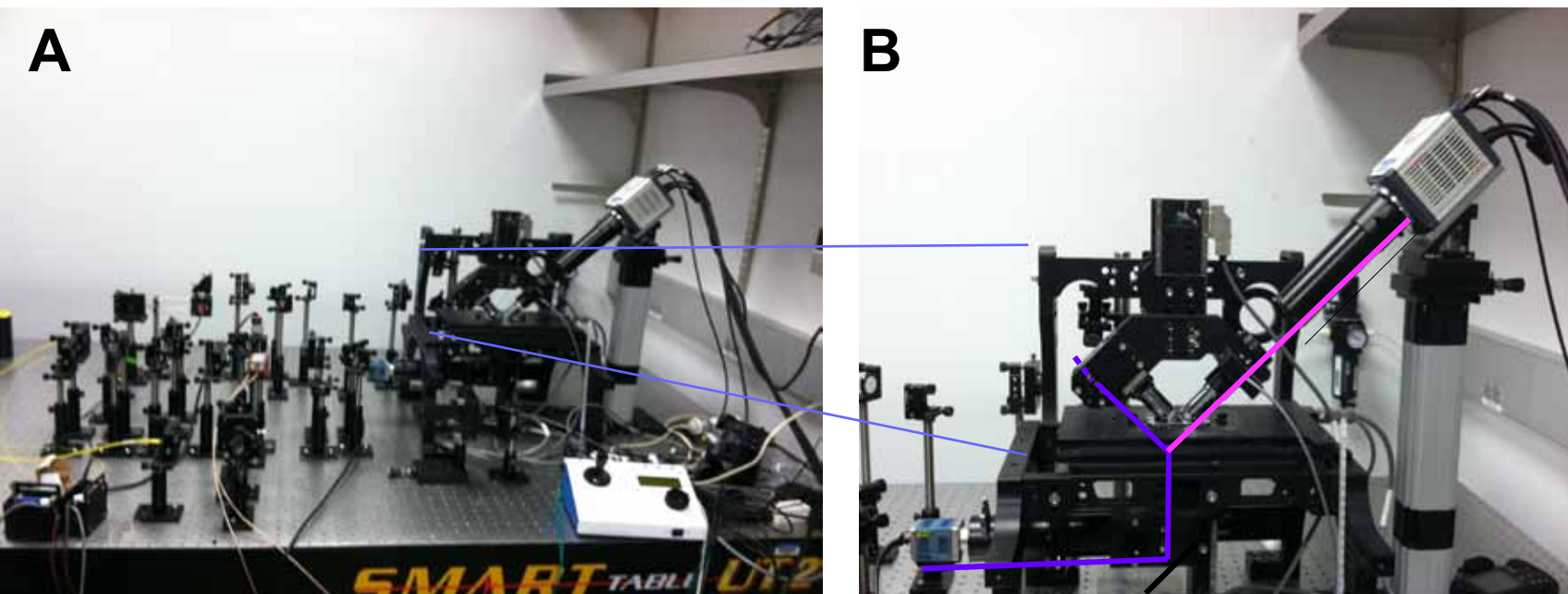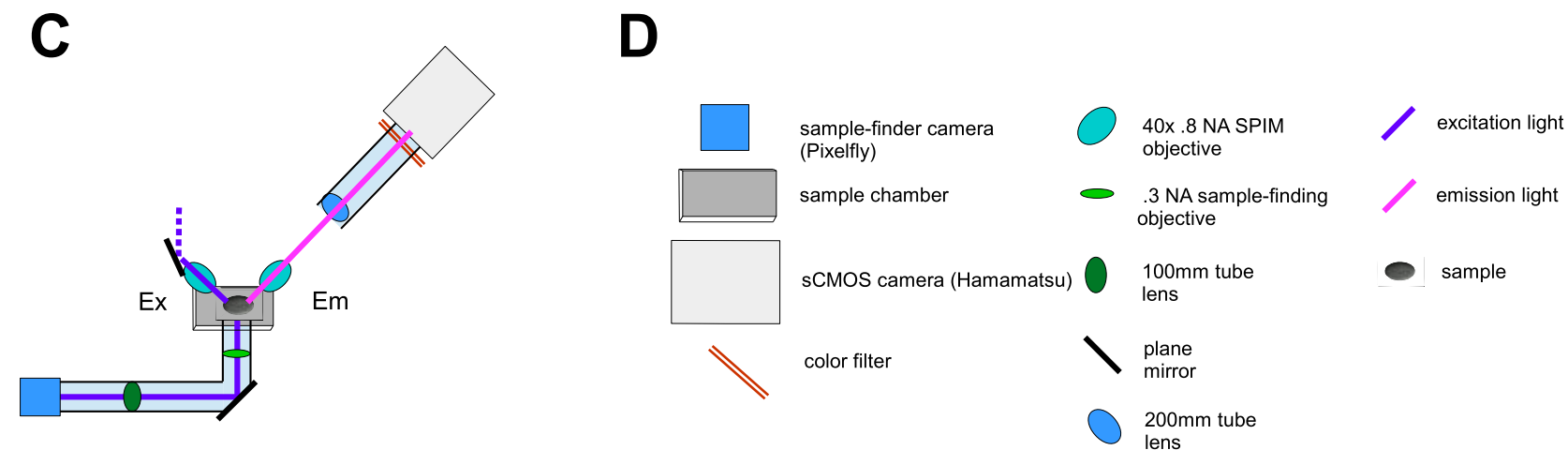

Supplement: S5 Fig — A diagram of the light path in the microscope body. Excitation light (purple) flows down through the excitation objective (“Ex”) to the sample, producing fluorescence (magenta), which is collected by the emission objective (“Em”) and conveyed to a Hamamatsu SCMOS camera. A second lens system and camera on the bottom axis, imaging at low NA, provides the capacity to locate samples for imaging. A) Front view of microscope. B) Overlay of light path on microscope body. C) Diagram of light path and optical components. D) Legend for B, C. (PDF) [file pone.0194861.s005.pdf]
